# Ventricular Dysfunction in Patients with Acute Coronary Syndrome Undergoing Coronary Surgical Revascularization: Prognostic Impact on Long-Term Outcomes

**DOI:** 10.1371/journal.pone.0168634

**Published:** 2016-12-22

**Authors:** Batric Popovic, Nelly Agrinier, Damien Voilliot, Mazen Elfarra, Jean Pierre Villemot, Pablo Maureira

**Affiliations:** 1 CHU Nancy, Département de Cardiologie, Nancy, France; 2 CHU Nancy, Epidémiologie et Evaluation Cliniques, Nancy, France; 3 CHU Nancy, Service de chirurgie des maladies cardiovasculaires et transplantations, Nancy, France; University of Milano, ITALY

## Abstract

**Background:**

Patients with non-ST elevation acute coronary syndrome complicated by left ventricular dysfunction (LVEF) are a poor prognosis group. The aim of our study was to assess the short and long term LEVF prognostic value in a cohort of NSTE-ACS patients undergoing surgical revascularization.

**Methods:**

We performed elective and isolated CABG on a cohort of 206 consecutive patients with LVEF≤0.40 complicating acute coronary syndrome. The case cohort was compared with a cohort of controls (LVEF>0.40) randomly selected (2:1) among patients who underwent the procedure during this period.

**Results:**

The Kaplan-Meier 5-year estimated survival rates for patients in the low and normal LVEF groups were 70.8% (95% confidence interval CI: 64.2–77.4) and 81.7% (95%CI: 77.8–85.6), respectively. A low LVEF was associated with both a higher all-cause (HR [95%CI] = 1.84[1.18–2.86]) and a higher cardiovascular mortality (HR = 2.07 [1.27–3.38]) during the first 12 months of follow-up. After adjustment for potential confounders, a low LVEF remained associated with a higher cardiovascular mortality only (1.87[1.03–3.38]) during the first 12 months of follow-up. After 12 months of follow-up, a low LVEF was no more associated with all-cause, nor cardiovascular mortality.

**Conclusion:**

Patients with low LVEF might require more intensive care than patients with normal LVEF during the year after the surgical procedure, but once the first postoperative year over, the initial low LVEF was no more associated with long term mortality.

## Introduction

Heart failure is one of the most frequent and severe complications of acute coronary syndrome [1]. Both congestive heart failure and left ventricular dysfunction are well established predictors of mortality in this population [2,3].

Severe left ventricular (LV) dysfunction caused by extensive coronary artery disease usually carries a poor prognosis, although surgical revascularization is thought to be the most effective treatment strategy in these patients [4, 5].

Despite these observations, Non-ST-Segment Elevation Acute Coronary Syndrome (NSTE-ACS) patients with left ventricular dysfunction less frequently receive evidence-based therapies including PCI or surgical revascularization [6] and, in most of the time, this high risk sub-group is underrepresented in trials concerning surgical revascularization in NSTE-ACS [7] or heart failure patients [8].

Moreover, all cardiac surgery risk- stratification models (Euroscore, STS score…) highlight LVEF as a major determinants of periprocedural mortality [9,10] but long term surgical results in this context are not usually emphasized enough.

The aim of our report was to assess the short and long term LEVF prognostic value in a cohort of NSTE-ACS patients undergoing surgical revascularization.

## Materials and Methods

### Study design

This study was an observational retrospective cohort of patients undergoing CABG exposed to low LVEF, who were compared to a concomitant cohort of patients with normal LVEF, recruited during the same period and in the same department of our university hospital.

From April 1996 to December 2008, 4210 patients with ACS underwent isolated CABG at our university center. Patients were eligible if they presented with a NSTE-ACS, i.e. if they had at least 10 minutes of ischemic symptoms at rest and presented with one of the following additional risk indicators: new ST-segment depression or transient elevation ≥1 mm or elevated biomarkers of myonecrosis (troponin Ic), and if they underwent CABG in our university hospital. Exclusion criteria were ST elevation coronary syndrome, cardiogenic shock, concomitant repair/replacement of valve, cardiac rupture, ventricular aneurysm or ascending aortic aneurysm.

From this group, we identified 1400 patients with ACS without ST-segment elevation (NSTE-ACS) at admission and among those patients 206 patients had a LVEF≤ 40% (case group). Patients in the case group were then compared to a control group of patients who were randomly selected from the patients with preserved LVEF who underwent CABG during the same period, and paired to the cases on the date of surgery using the nearest neighbors.

Control group sample size was calculated to identify risk factors with a type I error set at 1% to allow for multiple tests procedures, and a power of 80%. A randomization ratio of 2:1 was needed to fulfill the statistical requirements. A preoperative transthoracic two-dimensional echocardiography was performed in all patients. Left ventricular chamber dilatation was defined by a left ventricular diastolic diameter >54 mm and >60 mm in women and men, respectively [11].

CABG was performed using standard on-pump or off-pump bypass techniques at the discretion of the operating surgeon. Myocardial preservation during cardiopulmonary bypass involved normothermic, intermittent, anterograde and retrograde blood cardioplegia.

### Definitions of terms and data collection

Perioperative myocardial infarction (i.e., within 7 days after intervention) was defined as a creatine kinase-MB ≥5 times the upper limit of normal, with new Q waves in 2 contiguous leads on the postoperative electrocardiogram or the new development of bundle branch block. After this postoperative period, myocardial infarction was defined as the presence of new pathologic Q waves or the new development of left bundle branch block with increased cardiac marker levels (i.e., creatine kinase-MB ≥ 3 times the upper limit of normal). Cardiovascular death included death resulting from an acute myocardial infarction, hospitalization for heart failure, stroke, pulmonary embolism or digestive ischemia.

Patients did not undergo systematic control angiography during the follow-up period.

The endpoints were all-cause and cardiovascular mortality. Follow-up was performed through comprehensive questionnaires and by telephone with the patient’s personal physician. If death occurred, the patient’s physician or appropriate hospital record department were interviewed to document the cause. The retrospective study of patients’ files was approved by the Commission Nationale Informatique et Libertés (CNIL), in keeping with French law for single-center usual care observational studies.

### Statistical analysis

Baseline characteristics of the cases and control patients were described and compared. Continuous variables are presented as mean ± SD or median and interquartile range, and compared using a Student t-test or Wilcoxon test, according to conditions of application.

Categorical data are presented as numbers and percentages, and compared using a χ^2^ or Fisher’s exact test, according to conditions of application. We assessed the prognostic value of low LVEF, considering it as a time dependent variable, by the use of hazard ratios (HR). Crude and adjusted HR for all-cause and cardiovascular mortality were calculated using Cox semi-parametric regression models. Baseline characteristics associated with all-cause or cardiovascular mortality were entered into a stepwise logistic regression model with the significance level to enter set at 0.2 and the significance level to stay set at 0.05 to calculate adjusted LVEF HR. The significance level was set at 0.05. All statistical analyses were performed using SAS V.9.3 software (Cary, North Carolina, USA).

## Results

Baseline characteristics and perioperative course data are described in Tables 1 and 2.

**Table 1:** Clinical and demographic characteristics of the study groups.

	Low LVEF	Normal LVEF	
	n = 206	n = 412	
Age, years median (Inter quartile range)	66 [57 73]	65 [56–73]	0.7
Female sex	46 (22.3%)	64 (15.6%)	0.04
Hypertension	124 (62%)	261 (67.8%)	0.16
Diabetes mellitus	90 (45%)	119 (29.8%)	<0.001
Hypercholesterolemia *	123(61.5%)	290 (72.3%)	<0.001
History of smoking	126 (63%)	24 0 (59.7%)	0.4
Body mass index (kg/m^2^)	27 ±4.4	27 ±4.3	0.9
Other comorbid conditions			
chronic renal insufficiency †	21 (10.5%)	45 (11.3%)	0.8
COPD ‡	31 (15.5%)	48 (12%)	0.23
Peripheral artery disease	86 (41.7%)	122 (29.6%)	<0.01
Prior ischemic cardiopathy	130 (63.1%)	150 (36.4%)	<0.0001
Previous PCI §	69 (33.5%)	94 (22.8%)	<0.001
euroSCORE logistic (%) #	11 (6–15)	4 (3–6)	<0.001
Grace Score	179±23	140±28	<0.001
Symptoms: NYHA class			<0.0001
NYHA class I	46 (22.3%)	370 (89.8%)	
NYHA class II	63 (30.6%)	31 (7.5%)	
NYHA class III/IV	97 (47.1%)	11 (2.7%)	
Angiographic parameters			<0.001
three coronary arteries narrowed (≥ 70%)	137 (66.5%)	223 (54.3%)	
left main disease	27.5%	40%	
Mitral insufficiency			<0.001
grade 1	63 (30.5%)	56 (13.6%)	
grade 2	34 (16.5%)	16 (3.9%)	
grade 3/4	6 (2.9%)	0 (0%)	
Left ventricular chamber dilatation	24,4%	5%	<0.001
LVEF (%) ||	35 (30 40)	60 (50–60)	<0.0001

data presented as median and interquartile range or as mean ±SD

* Previously documented diagnosis of dyslipidemia or new diagnosis for a total cholesterol >200 mg/dl,

^†^ Creatinine clearance<40 ml/min,

^‡^ COPD: chronic obstructive pulmonary disease,

^§^ PCI: percutaneous coronary intervention,

^#^ euroSCORE: European System for Cardiac Operative Risk Evaluation score

^||^ LVEF: left ventricular ejection fraction

**Table 2:** Perioperative Data.

	Low LVEF	Normal LVEF	
	n = 206	n = 412	
On-pump surgery	28 (13.6%)	42 (10.2%)	0.2
Complete coronary revascularization	176 (85.4%)	311 (75.9%)	0.11
Extracorporeal circulation time (min)	80 (62 103)	77 (61 97)	0,4
Ventilation time < 24 hours	146 (73,4%)	331 (83,2%)	<0,005
IABP *	34(16.6%)	24 (5.8%)	<0.0001
GP2b3a inhibitors	28 (13.6%)	53 (12.9%)	0.8
Vasopressors or inotropic drugs (>24 hours)	102 (49,8%)	110 (26,7%)	<0,001
Transfusion (red blood cell units ≥3)	148 (74%)	322 (78.9%)	0.2

* intraaortic balloon counterpulsation

During the median [interquartile range] 65[22–100] months follow-up, a total of 166 died, of whom 78 in the case group and 88 in the control group. Among these deaths, 107 had a cardiovascular cause, 51 in the case group and 56 in the control group. At a six-month follow-up, a LVEF value was obtained in 93% (n = 383) and 80% of cases (n = 165) in the normal- and low-LVEF groups, respectively. Compared with the preoperative LVEF estimation, the six-month LVEF value in the low-LVEF group was improved in 59% of cases (n = 97) and unchanged or worsened in 41% of cases (n = 68), and a LVEF of >45% was achieved in 38% of cases (n = 63). A decrease in mitral regurgitation at 6 months was observed in only 50% cases.

**Fig 1:**
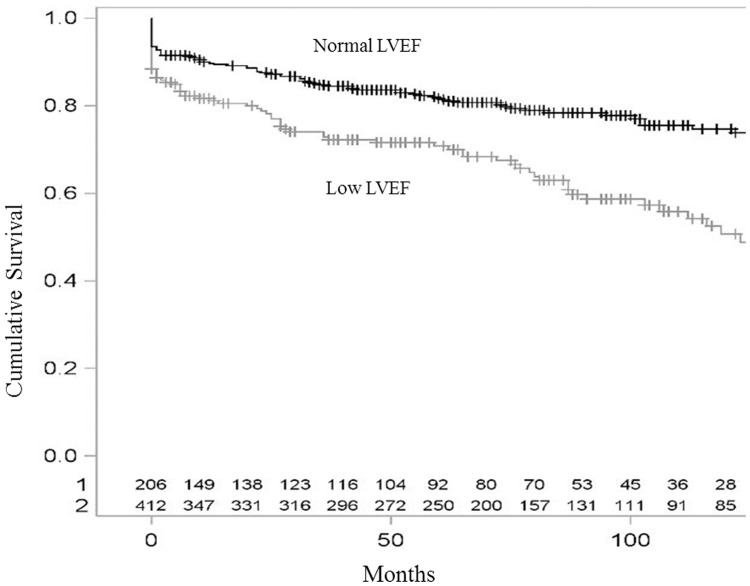
Kaplan-Meier overall survival curves for NSTE-ACS patients according to normal and low LVEF.

**Fig 2:**
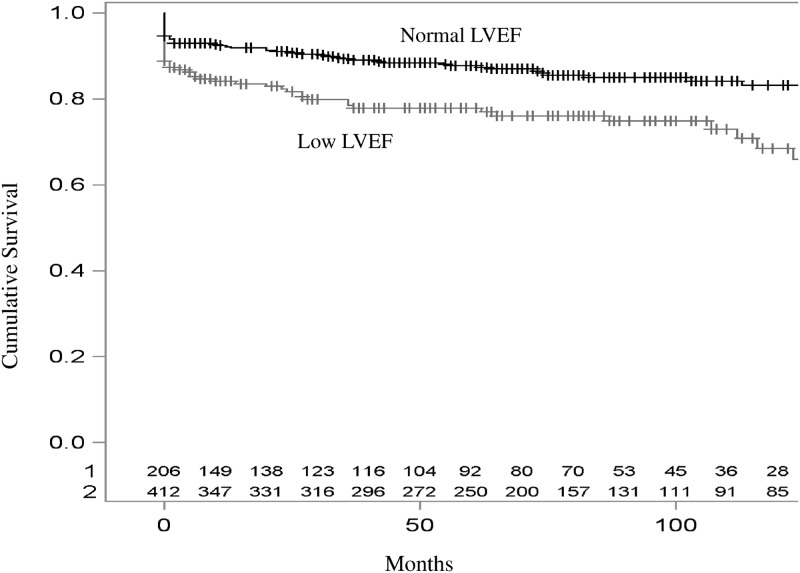
Kaplan-Meier cardiovascular survival curves for NSTE-ACS patients according to normal and low LVEF.

The Kaplan-Meier 5-year estimated survival rates for patients in the normal- and low-LVEF groups were 70.8% (95% confidence interval (CI): 64.2–77.4,) and 81.7% (95%CI: 77.8–85.6,), respectively. Figs 1 and 2 show Kaplan-Meier overall and cardiovascular survival curves for NSTE-ACS patients according to normal and low LVEF. As shown in Tables 3 and 4, baseline characteristics that were also associated (p<0.05) with an impaired all-cause (or cardiovascular) mortality were advanced age, lower BMI, chronic renal disease, past ischemic cardiomyopathy, polyvascular disease, and three-vessel disease. (advanced age, female sex, lower BMI, chronic renal condition, polyvascular disease, three-vessel disease, and supraventricular arrhythmia).

**Table 3:** Impact of baseline characteristics on all-cause mortality.

	Crude HR	95%CI	Adjusted HR^£^	95%CI
**Sociodemographics**				
Female	1.27	[0.86–1.88]		
Age(year)	1.07*	[1.05–1.09]	1.07	[1.05–1.09]
Body mass index (kg/m^2^)	0.95*	[0.91–0.99]		
**Medical history**				
Diabetes mellitus	1.00	[0.72–1.39]		
COPD †	1.33	[0.86–2.08]		
Hypertension	1.32*	[0.93–1.87]		
Chronic renal disease	2.95*	[2.00–4.34]	1.73	[1.10–2.72]
Dyslipidaemia	0.78*	[0.56–1.09]		
Smoker	0.82	[0.60–1.13]		
Ischemic cardiomyopathy	1.48*	[1.09–2.00]		
Past PCI ‡	0.99	[0.70–1.40]		
Polyvascular disease	2.05*	[1.51–2.80]	1.54	[1.06–2.25]
**Clinical characteristics**				
three-vessel disease	1.61*	[1.17–2.23]		
Supraventricular arrhythmia	1.26*	[0.90–1.76]		
Low LVEF §				
< 12 months	1.84	1.18–2.86	1.54	0.89–2.65
≥ 12 months	1.25	[0.68–2.31]	1.72	[0.83–3.58]

Note:

* p<0.20 in bivariate analyses,

^**£**^ Cox semi proportional model for all-cause mortality including low LVEF as a time-dependent variable and using a stepwise selection process sle = 0.2 sls = 0.05 to adjust for confounders

^†^ COPD: chronic obstructive pulmonary disease,

^‡^ PCI: percutaneous coronary intervention,

^§^ LVEF: left ventricular ejection fraction

**Table 4:** Impact of baseline characteristics on cardiovascular mortality.

	*Crude HR*	*95%CI*	*Adjusted HR*^£^	*95%CI*
**Sociodemographics**				
Female	1.70*	[1.09–2.64]		
Age(year)	1.07*	[1.05–1.09]	1.07	[1.04–1.09]
Body mass index (kg/m^2^)	0.94*	[0.89–0.99]		
**Medical history**				
Diabetes mellitus	1.14	[0.76–1.72]		
COPD †	1.03	[0.56–1.89]		
Hypertension	1.29	[0.83–2.00]		
Chronic renal disease	3.29*	[2.07–5.24]	1.91	[1.10–3.32]
Dyslipidaemia	0.74*	[0.49–1.11]		
Smoker	0.74*	[0.49–1.10]		
Ischemic cardiomyopathy	1.34*	[0.92–1.96]		
Past PCI ‡	1.00	[0.65–1.54]		
Polyvascular disease	1.85*	[1.26–2.71]	1.63	[1.01–2.64]
**Clinical characteristics**				
Three-vessel disease disease	1.72*	[1.14–2.59]		
Supraventricular arrhythmia	1.62*	[1.09–2.41]		
Low LVEF §				
< 12 months	2.07	[1.27–3.38]	1.87	[1.03–3.38]
≥ 12 months	0.99	[0.45–2.15]	1.24	[0.49–3.17]

Note:

* p<0.20 in bivariate analyses,

^**£**^ Cox semi proportional model for all-cause mortality including low LVEF as a time-dependent variable and using a stepwise selection process sle = 0.2 sls = 0.05 to adjust for confounders

^†^ COPD: chronic obstructive pulmonary disease,

^‡^ PCI: percutaneous coronary intervention,

^§^ LVEF: left ventricular ejection fraction

A low LVEF was associated with both a higher all-cause (HR [95%CI] = 1.84 [1.18–2.86] and a higher cardiovascular mortality (HR [95%CI] = 2.07 [1.27–3.38]) during the first 12 months of follow-up. After adjustment for potential confounders, a low LVEF remained associated with a higher cardiovascular mortality only (HR [95%CI] = 1.87 [1.03–3.38]) during the first 12 months of follow-up. After 12 months of follow-up, a low LVEF was no more associated with all-cause, nor cardiovascular mortality (Table 4).

## Discussion

Our study is one of the rare observational reports concerning the high risk ACS patients subgroup with left ventricular dysfunction. This high risk sub group is often underrepresented: in Stich trial evaluating the CABG in left ventricular dysfunction, about 5% of patients had an angina functional class III or IV and no ACS as initial clinical presentation was specified [8]. When a large scale as ACUITY trial compared the results surgical versus PCI for multivessel disease in ACS patients, patients with left ventricular dysfunction represented also a limited number of patients [7]. Moreover, Ranasinghe et al presented temporal trends in last decade concerning medications and revascularization procedures in ACS patients with chronic heart failure: while the rate of PCI revascularization tended to increase, there was no change in CABG group [6].

Our retrospective report gives us an opportunity to insist for a broader application of surgical revascularization in this high-risk group with multivessel coronary disease Low LVEF represents in our study an independent predictor of cardiovascular mortality during the first 12 months of follow-up. The cause of this higher mortality rate is likely to be multifactorial and may be related to a decreased ability to tolerate hemodynamic or ischemic complications in the setting of a CABG. This result corroborates the higher rate of adverse events observed during the periprocedural period with a higher proportion of impaired hemodynamic conditions indicated by a higher use of vasopressors/inotropic drugs, IABP and impaired renal function.

It is not surprising that NSTE ACS patients with low LVEF who underwent surgical revascularization are at higher cardiovascular risk, with more cardiac risk factors, comorbidities, and carry a greater burden of pre-existing cardiac disease. Nearly 50% showed evidence of heart failure (Killip class 3 or 4) at presentation and had substantially higher risk of in-hospital death measured as by the GRACE risk score [12].

On the other hand, Steg et al. demonstrated that ACS patients with heart failure are undertreated [1, 13] with a longer delay between admission and revascularization, a reduced frequency of revascularization and lower evidence-based medicine treatment usage at discharge in this high risk subgroup [1, 13, 14]. Moreover, as well as pharmacological treatment, the revascularization strategy implemented is a strong determinant of subsequent cardiovascular events, and the management of culprit lesion is still debated [2].

The main observation in our analysis concerns the long term outcomes in patients with low LVEF. After adjustment for potential confounders, a low LVEF at admission is no more associated with all cause or cardiovascular mortality after the first year of follow-up. Late survival, although markedly reduced when compared with patients with no decrease in LVEF, remains relatively high, with estimated survival rates of 70.8% at five years in our study.

In view of the poor prognosis of left ventricular dysfunction complicating ACS, it seems reassuring to consider that myocardial revascularization could prevent in some cases the left ventricular remodeling and improving left ventricular function at long term outcomes. Although several trials have previously provided the benefit of early revascularization of NSTE-ACS patients regardless of the presence of clinical heart failure or left ventricular dysfunction [15–17], Ranasinghe et al. suggests that most of the improvement in post ACS mortality over the past decades has occurred specifically in patients with chronic heart failure [6].

The prognosis of coronary revascularization is largely influenced by the reduction of ischemia and the preservation of the remaining myocardium leading to the improvement of postoperative LVEF. This benefit for LVEF is manifested especially in patients with ischemic but viable myocardium who subsequently underwent revascularization [8, 18]. However, the lack of correlation between myocardial viability and benefit from CABG in the Stich trial indicates that myocardial viability alone should not be the deciding factor in selecting the best strategy for these patients [18]. Other structural predictive parameters, such as left ventricular volumes and ischemic mitral regurgitation, associated with decreased survival are essential for interventional planning [19, 20].

In summary, the low LVEF represents only a transient predictive factor of cardiovascular mortality because of its progressive improvement during the first year of management. Initial eGFR [21, 22] and peripheral artery disease [23] are two others strong predictive factors of cardiovascular mortality in this population during the overall follow up. These factors should be taken into account to improve the risk prediction of this population and influence the revascularization strategy.

## Conclusion

Our retrospective report gives us an opportunity to insist for a broader application of surgical revascularization in this high-risk group with multivessel coronary disease.

Patients with left ventricular dysfunction complicating acute coronary syndrome represent a high risk population during the perioperative period and continuous efforts to improve care for these high-risk patients are important because once the first postoperative year is over, the initial low LVEF is no more associated with all-cause mortality. Heart team decision concerning the revascularization of these high risk patients should take into account of these results.
